# [μ-Bis(diphenyl­arsino)methane-1:2κ^2^ *As*:*As*′][(4-bromo­phen­yl)diphenyl­phosphine-3κ*P*]nona­carbonyl-1κ^3^ *C*,2κ^3^ *C*,3κ^3^ *C*-*triangulo*-tri­ruthenium(0) chloro­form 0.3-solvate

**DOI:** 10.1107/S1600536809055287

**Published:** 2010-01-16

**Authors:** Omar bin Shawkataly, Imthyaz Ahmed Khan, Chin Sing Yeap, Hoong-Kun Fun

**Affiliations:** a Chemical Sciences Programme, School of Distance Education, Universiti Sains Malaysia, 11800 USM, Penang, Malaysia; b X-ray Crystallography Unit, School of Physics, Universiti Sains Malaysia, 11800 USM, Penang, Malaysia

## Abstract

The asymmetric unit of the title *triangulo*-triruthenium compound, [Ru_3_(C_25_H_22_As_2_)(C_18_H_14_BrP)(CO)_9_]·0.3CHCl_3_, contains one mol­ecule of the *triangulo*-triruthenium complex and one partially occupied disordered chloro­form solvent mol­ecule. The bis­(diphenyl­arsino)methane ligand bridges an Ru—Ru bond and the monodentate phosphine ligand bonds to the third Ru atom. Both the arsine and phosphine ligands are equatorial with respect to the Ru_3_ triangle. In addition, each Ru atom carries one equatorial and two axial terminal carbonyl ligands. The phosphine-substituted benzene rings make dihedral angles of 67.5 (3), 76.1 (3) and 78.1 (3)° with each other. The dihedral angles between the two benzene rings are 79.0 (4) and 81.4 (3)° for the two diphenyl­arsino groups. In the crystal packing, the mol­ecules are linked into chains along the *a* axis by inter­molecular C—H⋯O hydrogen bonds.

## Related literature

For general background to *triangulo*-triruthenium derivatives, see: Bruce *et al.* (1985 ▶, 1988*a* ▶,*b* ▶). For related structures, see: Shawkataly *et al.* (1998 ▶, 2004 ▶, 2009 ▶). For the synthesis of μ-bis­(diphenyl­arsino)methane­deca­carbonyl­triruthenium(0), see: Bruce *et al.* (1983 ▶).
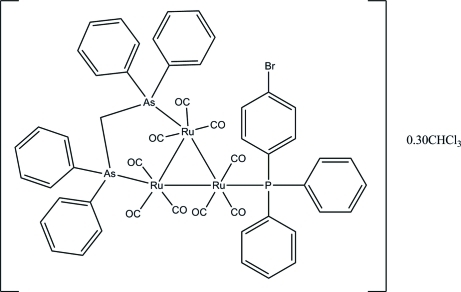


## Experimental

### 

#### Crystal data

[Ru_3_(C_25_H_22_As_2_)(C_18_H_14_BrP)(CO)_9_]·0.3CHCl_3_
*M* *_r_* = 1404.55
Monoclinic, 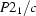

*a* = 13.2415 (2) Å
*b* = 16.9463 (3) Å
*c* = 25.2224 (4) Å
β = 91.831 (1)°
*V* = 5656.88 (16) Å^3^
*Z* = 4
Mo *K*α radiation
μ = 2.78 mm^−1^
*T* = 296 K
0.26 × 0.26 × 0.16 mm

#### Data collection

Bruker SMART APEXII CCD area-detector diffractometer
Absorption correction: multi-scan (*SADABS*; Bruker, 2005 ▶) *T*_min_ = 0.537, *T*_max_ = 0.673
63188 measured reflections
16521 independent reflections
10783 reflections with *I* > 2σ(*I*)
*R*_int_ = 0.042

#### Refinement

*R*[*F* ^2^ > 2σ(*F* ^2^)] = 0.048
*wR*(*F* ^2^) = 0.169
*S* = 1.08
16521 reflections
677 parameters
6 restraints
H-atom parameters constrained
Δρ_max_ = 1.41 e Å^−3^
Δρ_min_ = −1.35 e Å^−3^

### 

Data collection: *APEX2* (Bruker, 2005 ▶); cell refinement: *SAINT* (Bruker, 2005 ▶); data reduction: *SAINT*; program(s) used to solve structure: *SHELXTL* (Sheldrick, 2008 ▶); program(s) used to refine structure: *SHELXTL*; molecular graphics: *SHELXTL*; software used to prepare material for publication: *SHELXTL* and *PLATON* (Spek, 2009 ▶).

## Supplementary Material

Crystal structure: contains datablocks global, I. DOI: 10.1107/S1600536809055287/rz2406sup1.cif

Structure factors: contains datablocks I. DOI: 10.1107/S1600536809055287/rz2406Isup2.hkl

Additional supplementary materials:  crystallographic information; 3D view; checkCIF report

## Figures and Tables

**Table 1:** Hydrogen-bond geometry (Å, °)

*D*—H⋯*A*	*D*—H	H⋯*A*	*D*⋯*A*	*D*—H⋯*A*
C23—H23*A*⋯O2^i^	0.93	2.59	3.180 (8)	122

Symmetry code: (i) 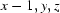.

**Fig. 1.**
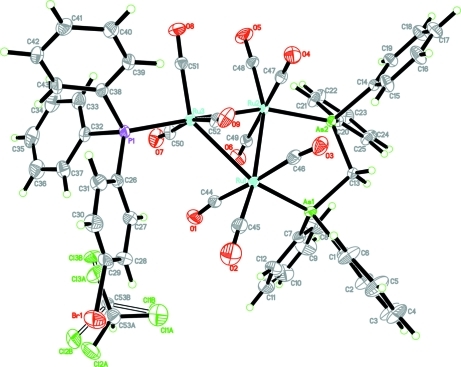
The molecular structure of the title compound with 20% probability ellipsoids for non-H atoms. All disordered components are shown.

**Fig. 2.**
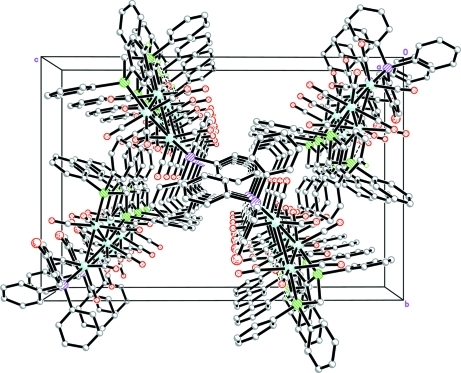
The crystal packing of the title compound, viewed down the a axis, showing the molecules linked into chains along a axis. Hydrogen atoms not involved in the hydrogen-bonding (dashed lines) and the solvent molecules have been omitted for clarity.

## supplementary crystallographic information

### Comment

*Triangulo*-triruthenium clusters are known for their interesting structural variations and related catalytic activity. A large number of substituted derivatives, Ru_3_(CO)_12-n_*L*_n_ (*L* = group 15 ligand) have been reported (Bruce *et al.*, 1985, 1988*a*,*b*). As part of our study on the substitution of transition metal-carbonyl clusters with mixed-ligand complexes, we have published several structures of *triangulo*-triruthenium-carbonyl clusters containing mixed P/As and P/Sb ligands (Shawkataly *et al.*, 1998, 2004, 2009). Herein we report the synthesis and structure of title compound.

The asymmetric unit consists of one molecule of the *triangulo*-triruthenium complex and a 30% partially occupied molecule of disordered chloroform solvent (Fig. 1). The bond lengths and angles of title compound are comparable to those found in its related structure (Shawkataly *et al.*, 2009). The bis(diphenylarsino)methane ligand bridges the Ru1—Ru2 bond and the monodentate phosphine ligand bonds to the Ru3 atom. Both the phosphine and arsine ligands are equatorial with respect to the Ru_3_ triangle. Additionally, each Ru atom carries one equatorial and two axial terminal carbonyl ligands. The phosphine-substituted benzene rings make dihedral angles (C26–C31/C32–C37, C26–C31/C38–C43 and C32–C37/C38–C43) of 67.5 (3), 76.1 (3) and 78.1 (3)° with each other respectively. The dihedral angles between the two benzene rings (C1–C6/C7–C12 and C14–C19/C20–C25) are 79.0 (4) and 81.4 (3)° for the two diphenylarsino groups respectively.

In the crystal packing (Fig. 2), the molecules are linked into chains along the *a* axis by intermolecular C23—H23A···O2 hydrogen bonds (Table 1).

### Experimental

All manipulations were performed under a dry, oxygen-free dinitrogen atmosphere using standard Schlenk techniques, all solvents were dried over sodium and distilled from sodium benzophenone ketyl under nitrogen. (4-Bromophenyl)diphenylphosphine (Maybridge) was used as received and µ-bis(diphenylarsino)methane-decacarbonyl-triruthenium(0) (Bruce *et al.*, 1983) were prepared by a reported procedure. The title compound was obtained by refluxing equimolar quantities of Ru_3_(CO)_10_(µ-Ph_2_AsCH_2_AsPh_2_) (105.5 mg, 0.1 mmol) and (4-bromophenyl)diphenylphosphine (34.12 mg, 0.1 mmol) in hexane under nitrogen atmosphere. Crystals suitable for X-ray diffraction were grown by slow solvent / solvent diffusion of CH_3_OH into CH_2_Cl_2_.

### Refinement

All hydrogen atoms were positioned geometrically and refined using a riding model with C—H = 0.93–0.97 Å and *U*_iso_(H) = 1.2 *U*_eq_(C). The chloroform molecule is disordered over two positions with the site occupancies fixed to 0.15 for both components and with SADI command for the final refinement. The C53A and C53B atoms were refined isotropically. The maximum and minimum residual electron density peaks of 1.41 and -1.35 e Å^-3^, respectively, were located 0.56 Å and 0.96 Å from the Cl2B and Br1 atoms, respectively.

### Crystal data

**Table:**

[Ru_3_(C_25_H_22_As_2_)(C_18_H_14_BrP)(CO)_9_]·0.3CHCl_3_	*F*(000) = 2742
*M**_r_* = 1404.55	*D*_x_ = 1.649 Mg m^−^^3^
Monoclinic, *P*2_1_/*c*	Mo *K*α radiation, λ = 0.71073 Å
Hall symbol: -P 2ybc	Cell parameters from 9923 reflections
*a* = 13.2415 (2) Å	θ = 2.2–27.8°
*b* = 16.9463 (3) Å	µ = 2.78 mm^−^^1^
*c* = 25.2224 (4) Å	*T* = 296 K
β = 91.831 (1)°	Block, purple
*V* = 5656.88 (16) Å^3^	0.26 × 0.26 × 0.16 mm
*Z* = 4	

### Data collection

**Table:**

Bruker SMART APEXII CCD area-detector diffractometer	16521 independent reflections
Radiation source: fine-focus sealed tube	10783 reflections with *I* > 2σ(*I*)
graphite	*R*_int_ = 0.042
φ and ω scans	θ_max_ = 30.1°, θ_min_ = 1.5°
Absorption correction: multi-scan (*SADABS*; Bruker, 2005)	*h* = −15→18
*T*_min_ = 0.537, *T*_max_ = 0.673	*k* = −23→23
63188 measured reflections	*l* = −34→35

### Refinement

**Table:**

Refinement on *F*^2^	Primary atom site location: structure-invariant direct methods
Least-squares matrix: full	Secondary atom site location: difference Fourier map
*R*[*F*^2^ > 2σ(*F*^2^)] = 0.048	Hydrogen site location: inferred from neighbouring sites
*wR*(*F*^2^) = 0.169	H-atom parameters constrained
*S* = 1.08	*w* = 1/[σ^2^(*F*_o_^2^) + (0.0909*P*)^2^ + 1.7904*P*] where *P* = (*F*_o_^2^ + 2*F*_c_^2^)/3
16521 reflections	(Δ/σ)_max_ < 0.001
677 parameters	Δρ_max_ = 1.41 e Å^−^^3^
6 restraints	Δρ_min_ = −1.35 e Å^−^^3^

### Special details

**Table:**

Geometry. All e.s.d.'s (except the e.s.d. in the dihedral angle between two l.s. planes) are estimated using the full covariance matrix. The cell e.s.d.'s are taken into account individually in the estimation of e.s.d.'s in distances, angles and torsion angles; correlations between e.s.d.'s in cell parameters are only used when they are defined by crystal symmetry. An approximate (isotropic) treatment of cell e.s.d.'s is used for estimating e.s.d.'s involving l.s. planes.
Refinement. Refinement of *F*^2^ against ALL reflections. The weighted *R*-factor *wR* and goodness of fit *S* are based on *F*^2^, conventional *R*-factors *R* are based on *F*, with *F* set to zero for negative *F*^2^. The threshold expression of *F*^2^ > σ(*F*^2^) is used only for calculating *R*-factors(gt) *etc*. and is not relevant to the choice of reflections for refinement. *R*-factors based on *F*^2^ are statistically about twice as large as those based on *F*, and *R*- factors based on ALL data will be even larger.

### Fractional atomic coordinates and isotropic or equivalent isotropic displacement parameters (Å^2^)

**Table:**

	*x*	*y*	*z*	*U*_iso_*/*U*_eq_	Occ. (<1)
Ru1	0.23647 (3)	0.31292 (2)	0.159957 (17)	0.03734 (11)	
Ru2	0.04996 (3)	0.23846 (2)	0.182974 (15)	0.03237 (10)	
Ru3	0.19834 (3)	0.15122 (2)	0.129329 (15)	0.03373 (11)	
As1	0.16719 (4)	0.44257 (3)	0.18017 (2)	0.03586 (13)	
As2	−0.01816 (3)	0.34651 (3)	0.234290 (19)	0.03180 (12)	
P1	0.31671 (10)	0.08260 (8)	0.07993 (5)	0.0391 (3)	
Br1	0.73998 (7)	0.26067 (7)	0.03555 (5)	0.1197 (4)	
O1	0.1908 (4)	0.3326 (3)	0.04092 (18)	0.0692 (13)	
O2	0.4533 (4)	0.3563 (4)	0.1392 (3)	0.128 (3)	
O3	0.2858 (4)	0.2977 (3)	0.2792 (2)	0.0750 (14)	
O4	0.1342 (4)	0.1548 (3)	0.28246 (18)	0.0709 (13)	
O5	−0.1249 (4)	0.1225 (3)	0.1762 (2)	0.0891 (17)	
O6	−0.0303 (3)	0.3221 (3)	0.08269 (17)	0.0646 (12)	
O7	0.0669 (4)	0.1772 (3)	0.02798 (19)	0.0770 (14)	
O8	0.0746 (3)	0.0079 (3)	0.15645 (19)	0.0663 (12)	
O9	0.3573 (3)	0.1471 (2)	0.21969 (19)	0.0634 (12)	
C1	0.2604 (4)	0.5220 (3)	0.2096 (2)	0.0463 (13)	
C2	0.2406 (5)	0.6013 (4)	0.2031 (3)	0.0702 (19)	
H2A	0.1840	0.6175	0.1832	0.084*	
C3	0.3065 (6)	0.6578 (4)	0.2265 (4)	0.089 (3)	
H3A	0.2934	0.7114	0.2222	0.107*	
C4	0.3881 (6)	0.6337 (5)	0.2549 (4)	0.088 (3)	
H4A	0.4315	0.6709	0.2703	0.106*	
C5	0.4079 (6)	0.5570 (5)	0.2613 (4)	0.096 (3)	
H5A	0.4644	0.5416	0.2815	0.115*	
C6	0.3448 (5)	0.4995 (4)	0.2380 (3)	0.080 (2)	
H6A	0.3604	0.4463	0.2419	0.096*	
C7	0.1009 (4)	0.5021 (3)	0.1233 (2)	0.0451 (12)	
C8	0.0047 (5)	0.5283 (5)	0.1246 (3)	0.076 (2)	
H8A	−0.0345	0.5171	0.1535	0.091*	
C9	−0.0355 (7)	0.5722 (6)	0.0823 (4)	0.105 (3)	
H9A	−0.1021	0.5894	0.0837	0.125*	
C10	0.0150 (8)	0.5901 (6)	0.0414 (4)	0.095 (3)	
H10A	−0.0150	0.6198	0.0141	0.115*	
C11	0.1142 (8)	0.5653 (5)	0.0379 (3)	0.093 (3)	
H11A	0.1517	0.5785	0.0086	0.111*	
C12	0.1568 (5)	0.5199 (4)	0.0791 (3)	0.0703 (19)	
H12A	0.2227	0.5015	0.0770	0.084*	
C13	0.0693 (4)	0.4407 (3)	0.2370 (2)	0.0373 (11)	
H13A	0.0273	0.4876	0.2343	0.045*	
H13B	0.1055	0.4423	0.2710	0.045*	
C14	−0.0395 (4)	0.3275 (3)	0.30969 (19)	0.0376 (11)	
C15	0.0424 (4)	0.3164 (4)	0.3438 (2)	0.0564 (16)	
H15A	0.1077	0.3197	0.3315	0.068*	
C16	0.0267 (5)	0.3004 (5)	0.3969 (2)	0.069 (2)	
H16A	0.0820	0.2927	0.4200	0.083*	
C17	−0.0679 (6)	0.2958 (4)	0.4156 (3)	0.0707 (19)	
H17A	−0.0770	0.2853	0.4513	0.085*	
C18	−0.1519 (5)	0.3068 (5)	0.3813 (3)	0.0681 (19)	
H18A	−0.2171	0.3037	0.3938	0.082*	
C19	−0.1358 (4)	0.3225 (4)	0.3283 (2)	0.0511 (14)	
H19A	−0.1909	0.3297	0.3050	0.061*	
C20	−0.1490 (3)	0.3869 (3)	0.21020 (19)	0.0368 (11)	
C21	−0.2132 (4)	0.3360 (4)	0.1828 (3)	0.0545 (15)	
H21A	−0.1924	0.2848	0.1755	0.065*	
C22	−0.3100 (4)	0.3616 (4)	0.1660 (3)	0.069 (2)	
H22A	−0.3531	0.3277	0.1471	0.083*	
C23	−0.3408 (4)	0.4376 (4)	0.1778 (3)	0.0614 (17)	
H23A	−0.4050	0.4548	0.1671	0.074*	
C24	−0.2765 (4)	0.4878 (3)	0.2054 (2)	0.0501 (14)	
H24A	−0.2968	0.5389	0.2132	0.060*	
C25	−0.1817 (4)	0.4617 (3)	0.2214 (2)	0.0428 (12)	
H25A	−0.1387	0.4958	0.2402	0.051*	
C26	0.4375 (4)	0.1316 (3)	0.0694 (2)	0.0433 (12)	
C27	0.4367 (5)	0.2075 (4)	0.0496 (2)	0.0574 (15)	
H27A	0.3755	0.2332	0.0428	0.069*	
C28	0.5284 (5)	0.2465 (4)	0.0394 (3)	0.0693 (19)	
H28A	0.5281	0.2968	0.0248	0.083*	
C29	0.6169 (5)	0.2092 (5)	0.0514 (3)	0.069 (2)	
C30	0.6201 (5)	0.1363 (5)	0.0732 (3)	0.077 (2)	
H30A	0.6817	0.1130	0.0827	0.092*	
C31	0.5310 (4)	0.0971 (4)	0.0813 (3)	0.0603 (16)	
H31A	0.5332	0.0461	0.0950	0.072*	
C32	0.2688 (4)	0.0565 (3)	0.0135 (2)	0.0468 (13)	
C33	0.1857 (5)	0.0077 (4)	0.0086 (3)	0.0665 (18)	
H33A	0.1581	−0.0131	0.0390	0.080*	
C34	0.1424 (5)	−0.0111 (5)	−0.0403 (4)	0.086 (3)	
H34A	0.0866	−0.0443	−0.0428	0.103*	
C35	0.1842 (7)	0.0212 (5)	−0.0873 (3)	0.088 (3)	
H35A	0.1560	0.0090	−0.1206	0.105*	
C36	0.2638 (7)	0.0686 (5)	−0.0828 (3)	0.084 (2)	
H36A	0.2910	0.0898	−0.1132	0.101*	
C37	0.3079 (5)	0.0874 (4)	−0.0327 (2)	0.0645 (17)	
H37A	0.3636	0.1207	−0.0305	0.077*	
C38	0.3574 (4)	−0.0136 (3)	0.1050 (2)	0.0433 (12)	
C39	0.3556 (4)	−0.0316 (4)	0.1587 (2)	0.0523 (14)	
H39A	0.3277	0.0044	0.1819	0.063*	
C40	0.3944 (5)	−0.1020 (4)	0.1784 (3)	0.0623 (17)	
H40A	0.3925	−0.1131	0.2145	0.075*	
C41	0.4354 (5)	−0.1553 (4)	0.1442 (3)	0.0685 (19)	
H41A	0.4647	−0.2015	0.1574	0.082*	
C42	0.4335 (5)	−0.1402 (4)	0.0897 (3)	0.071 (2)	
H42A	0.4572	−0.1777	0.0663	0.085*	
C43	0.3966 (5)	−0.0703 (4)	0.0715 (2)	0.0571 (16)	
H43A	0.3974	−0.0600	0.0353	0.069*	
C44	0.2033 (4)	0.3203 (3)	0.0846 (3)	0.0491 (14)	
C45	0.3714 (4)	0.3393 (4)	0.1466 (3)	0.0670 (19)	
C46	0.2647 (5)	0.2992 (3)	0.2354 (3)	0.0525 (14)	
C47	0.1078 (4)	0.1867 (3)	0.2453 (2)	0.0475 (13)	
C48	−0.0591 (4)	0.1669 (3)	0.1799 (3)	0.0513 (14)	
C49	0.0046 (4)	0.2904 (3)	0.1191 (2)	0.0431 (12)	
C50	0.1146 (4)	0.1708 (4)	0.0662 (2)	0.0481 (13)	
C51	0.1244 (4)	0.0604 (3)	0.1463 (2)	0.0428 (12)	
C52	0.2949 (4)	0.1556 (3)	0.1875 (2)	0.0442 (12)	
Cl1A	0.3768 (15)	0.4277 (14)	−0.0348 (9)	0.097 (6)	0.15
Cl2A	0.5082 (16)	0.3887 (11)	−0.1025 (9)	0.118 (7)	0.15
Cl3A	0.326 (2)	0.3053 (11)	−0.0909 (11)	0.102 (7)	0.15
C53A	0.393 (2)	0.382 (2)	−0.0880 (16)	0.073 (13)*	0.15
H53A	0.3597	0.4163	−0.1145	0.088*	0.15
Cl1B	0.3361 (12)	0.4365 (13)	−0.0503 (7)	0.084 (5)	0.15
Cl2B	0.4565 (12)	0.3670 (9)	−0.1348 (7)	0.085 (5)	0.15
Cl3B	0.3069 (15)	0.2742 (9)	−0.0856 (6)	0.064 (4)	0.15
C53B	0.348 (2)	0.3668 (12)	−0.0994 (11)	0.042 (8)*	0.15
H53B	0.2971	0.3845	−0.1260	0.051*	0.15

### Atomic displacement parameters (Å^2^)

**Table:**

	*U*^11^	*U*^22^	*U*^33^	*U*^12^	*U*^13^	*U*^23^
Ru1	0.03017 (19)	0.0334 (2)	0.0485 (3)	−0.00255 (15)	0.00296 (16)	−0.00460 (18)
Ru2	0.03149 (19)	0.0291 (2)	0.0367 (2)	−0.00158 (14)	0.00485 (15)	−0.00434 (16)
Ru3	0.03338 (19)	0.0313 (2)	0.0368 (2)	0.00096 (15)	0.00430 (15)	−0.00422 (16)
As1	0.0338 (2)	0.0305 (3)	0.0433 (3)	−0.00346 (19)	0.0004 (2)	−0.0003 (2)
As2	0.0318 (2)	0.0296 (3)	0.0341 (3)	0.00008 (18)	0.00137 (19)	−0.0033 (2)
P1	0.0365 (6)	0.0405 (7)	0.0407 (7)	−0.0001 (5)	0.0085 (5)	−0.0065 (6)
Br1	0.0793 (6)	0.1379 (9)	0.1450 (9)	−0.0582 (6)	0.0501 (6)	−0.0405 (7)
O1	0.082 (3)	0.077 (3)	0.049 (3)	−0.009 (3)	0.007 (2)	0.007 (2)
O2	0.047 (3)	0.155 (6)	0.182 (7)	−0.041 (3)	0.029 (4)	−0.067 (5)
O3	0.106 (4)	0.060 (3)	0.058 (3)	0.003 (3)	−0.017 (3)	−0.005 (2)
O4	0.094 (4)	0.061 (3)	0.057 (3)	0.022 (3)	−0.002 (2)	0.015 (2)
O5	0.062 (3)	0.061 (3)	0.147 (5)	−0.029 (2)	0.029 (3)	−0.022 (3)
O6	0.054 (2)	0.085 (3)	0.054 (3)	0.009 (2)	−0.001 (2)	0.010 (2)
O7	0.090 (3)	0.082 (4)	0.058 (3)	0.016 (3)	−0.022 (3)	−0.008 (3)
O8	0.069 (3)	0.046 (3)	0.084 (3)	−0.013 (2)	0.006 (2)	0.007 (2)
O9	0.060 (3)	0.049 (3)	0.080 (3)	0.013 (2)	−0.027 (2)	−0.013 (2)
C1	0.039 (3)	0.040 (3)	0.059 (4)	−0.010 (2)	−0.001 (2)	−0.005 (3)
C2	0.065 (4)	0.042 (4)	0.102 (6)	−0.003 (3)	−0.022 (4)	−0.005 (4)
C3	0.077 (5)	0.042 (4)	0.146 (8)	−0.015 (3)	−0.027 (5)	−0.014 (4)
C4	0.064 (4)	0.060 (5)	0.139 (8)	−0.016 (4)	−0.027 (5)	−0.030 (5)
C5	0.083 (5)	0.062 (5)	0.138 (8)	−0.008 (4)	−0.059 (5)	−0.002 (5)
C6	0.071 (4)	0.040 (4)	0.126 (7)	−0.008 (3)	−0.044 (4)	0.002 (4)
C7	0.051 (3)	0.038 (3)	0.046 (3)	−0.004 (2)	−0.001 (2)	0.004 (2)
C8	0.058 (4)	0.095 (6)	0.074 (5)	0.013 (4)	0.001 (3)	0.038 (4)
C9	0.089 (6)	0.122 (8)	0.100 (7)	0.030 (5)	−0.021 (5)	0.050 (6)
C10	0.111 (7)	0.098 (7)	0.075 (6)	0.008 (6)	−0.026 (5)	0.043 (5)
C11	0.158 (9)	0.073 (5)	0.048 (4)	−0.024 (6)	0.005 (5)	0.019 (4)
C12	0.075 (4)	0.071 (5)	0.065 (4)	−0.006 (4)	0.008 (3)	0.018 (4)
C13	0.042 (3)	0.036 (3)	0.035 (3)	−0.003 (2)	0.002 (2)	−0.004 (2)
C14	0.044 (3)	0.033 (3)	0.036 (3)	−0.002 (2)	0.005 (2)	−0.003 (2)
C15	0.047 (3)	0.082 (5)	0.041 (3)	0.008 (3)	0.001 (2)	−0.002 (3)
C16	0.062 (4)	0.109 (6)	0.037 (3)	0.005 (4)	−0.005 (3)	0.009 (3)
C17	0.083 (5)	0.082 (5)	0.047 (4)	−0.008 (4)	0.013 (3)	0.007 (4)
C18	0.064 (4)	0.088 (5)	0.053 (4)	−0.008 (4)	0.015 (3)	0.005 (4)
C19	0.042 (3)	0.064 (4)	0.048 (3)	−0.004 (3)	0.001 (2)	0.004 (3)
C20	0.034 (2)	0.041 (3)	0.036 (3)	0.002 (2)	−0.0006 (19)	−0.003 (2)
C21	0.041 (3)	0.049 (3)	0.073 (4)	0.003 (2)	−0.007 (3)	−0.019 (3)
C22	0.036 (3)	0.073 (5)	0.097 (5)	0.004 (3)	−0.017 (3)	−0.028 (4)
C23	0.039 (3)	0.072 (4)	0.073 (4)	0.017 (3)	−0.009 (3)	0.000 (3)
C24	0.046 (3)	0.041 (3)	0.063 (4)	0.008 (2)	−0.006 (3)	−0.001 (3)
C25	0.040 (3)	0.038 (3)	0.049 (3)	−0.001 (2)	−0.004 (2)	−0.005 (2)
C26	0.038 (3)	0.053 (3)	0.039 (3)	−0.004 (2)	0.005 (2)	−0.009 (2)
C27	0.055 (3)	0.055 (4)	0.063 (4)	−0.004 (3)	0.013 (3)	−0.002 (3)
C28	0.072 (4)	0.063 (4)	0.074 (5)	−0.017 (3)	0.026 (4)	0.001 (3)
C29	0.045 (3)	0.089 (5)	0.075 (5)	−0.028 (3)	0.026 (3)	−0.027 (4)
C30	0.044 (3)	0.094 (6)	0.094 (6)	−0.013 (4)	0.012 (3)	−0.014 (5)
C31	0.046 (3)	0.066 (4)	0.070 (4)	0.001 (3)	0.001 (3)	0.000 (3)
C32	0.043 (3)	0.050 (3)	0.047 (3)	0.003 (2)	0.000 (2)	−0.015 (3)
C33	0.058 (4)	0.069 (4)	0.073 (5)	−0.003 (3)	0.002 (3)	−0.020 (4)
C34	0.061 (4)	0.085 (6)	0.110 (7)	−0.001 (4)	−0.023 (4)	−0.042 (5)
C35	0.108 (7)	0.086 (6)	0.068 (5)	0.016 (5)	−0.029 (5)	−0.029 (5)
C36	0.108 (6)	0.091 (6)	0.051 (4)	0.007 (5)	−0.012 (4)	−0.011 (4)
C37	0.065 (4)	0.080 (5)	0.049 (4)	0.003 (3)	0.002 (3)	−0.010 (3)
C38	0.040 (3)	0.039 (3)	0.051 (3)	0.003 (2)	0.010 (2)	−0.004 (2)
C39	0.057 (3)	0.046 (3)	0.054 (4)	0.008 (3)	0.008 (3)	−0.008 (3)
C40	0.077 (4)	0.050 (4)	0.061 (4)	0.011 (3)	0.012 (3)	0.003 (3)
C41	0.069 (4)	0.044 (4)	0.093 (5)	0.017 (3)	0.016 (4)	0.004 (4)
C42	0.079 (5)	0.049 (4)	0.087 (5)	0.016 (3)	0.034 (4)	−0.008 (4)
C43	0.070 (4)	0.050 (4)	0.053 (4)	0.014 (3)	0.018 (3)	−0.007 (3)
C44	0.041 (3)	0.048 (3)	0.058 (4)	−0.008 (2)	0.011 (3)	−0.004 (3)
C45	0.040 (3)	0.069 (4)	0.092 (5)	−0.011 (3)	0.007 (3)	−0.020 (4)
C46	0.060 (4)	0.037 (3)	0.060 (4)	0.006 (3)	−0.004 (3)	−0.001 (3)
C47	0.053 (3)	0.037 (3)	0.053 (4)	0.008 (2)	0.008 (3)	−0.003 (3)
C48	0.043 (3)	0.042 (3)	0.070 (4)	−0.005 (2)	0.014 (3)	−0.010 (3)
C49	0.035 (2)	0.051 (3)	0.044 (3)	0.000 (2)	0.001 (2)	−0.001 (3)
C50	0.049 (3)	0.051 (3)	0.044 (3)	0.007 (3)	0.000 (3)	−0.007 (3)
C51	0.041 (3)	0.041 (3)	0.046 (3)	0.002 (2)	0.001 (2)	0.000 (2)
C52	0.047 (3)	0.035 (3)	0.051 (3)	0.002 (2)	−0.002 (2)	−0.011 (2)
Cl1A	0.087 (13)	0.096 (12)	0.106 (14)	0.037 (11)	−0.019 (10)	0.010 (10)
Cl2A	0.130 (16)	0.092 (12)	0.136 (17)	0.018 (11)	0.087 (13)	0.028 (12)
Cl3A	0.117 (16)	0.054 (12)	0.135 (17)	0.003 (10)	−0.007 (12)	0.008 (11)
Cl1B	0.062 (9)	0.110 (13)	0.081 (11)	0.027 (9)	0.012 (8)	0.000 (9)
Cl2B	0.098 (11)	0.060 (8)	0.099 (11)	0.058 (8)	0.034 (8)	0.020 (8)
Cl3B	0.090 (9)	0.046 (9)	0.054 (7)	−0.010 (7)	−0.012 (6)	0.027 (7)

### Geometric parameters (Å, °)

**Table:**

Ru1—C45	1.883 (6)	C15—C16	1.388 (8)
Ru1—C44	1.940 (7)	C15—H15A	0.9300
Ru1—C46	1.942 (7)	C16—C17	1.354 (9)
Ru1—As1	2.4410 (6)	C16—H16A	0.9300
Ru1—Ru2	2.8495 (5)	C17—C18	1.400 (10)
Ru1—Ru3	2.8870 (6)	C17—H17A	0.9300
Ru2—C48	1.885 (6)	C18—C19	1.386 (8)
Ru2—C49	1.915 (6)	C18—H18A	0.9300
Ru2—C47	1.935 (6)	C19—H19A	0.9300
Ru2—As2	2.4323 (6)	C20—C25	1.371 (7)
Ru2—Ru3	2.8366 (5)	C20—C21	1.382 (7)
Ru3—C51	1.880 (5)	C21—C22	1.405 (8)
Ru3—C52	1.916 (6)	C21—H21A	0.9300
Ru3—C50	1.940 (6)	C22—C23	1.388 (9)
Ru3—P1	2.3423 (13)	C22—H22A	0.9300
As1—C7	1.941 (5)	C23—C24	1.376 (8)
As1—C1	1.957 (5)	C23—H23A	0.9300
As1—C13	1.964 (5)	C24—C25	1.379 (7)
As2—C20	1.942 (5)	C24—H24A	0.9300
As2—C14	1.958 (5)	C25—H25A	0.9300
As2—C13	1.972 (5)	C26—C27	1.381 (8)
P1—C38	1.823 (6)	C26—C31	1.393 (8)
P1—C32	1.828 (6)	C27—C28	1.412 (8)
P1—C26	1.828 (5)	C27—H27A	0.9300
Br1—C29	1.902 (6)	C28—C29	1.358 (10)
O1—C44	1.129 (7)	C28—H28A	0.9300
O2—C45	1.143 (7)	C29—C30	1.352 (11)
O3—C46	1.132 (7)	C30—C31	1.375 (9)
O4—C47	1.130 (7)	C30—H30A	0.9300
O5—C48	1.153 (7)	C31—H31A	0.9300
O6—C49	1.149 (6)	C32—C33	1.380 (8)
O7—C50	1.140 (7)	C32—C37	1.390 (8)
O8—C51	1.142 (6)	C33—C34	1.381 (10)
O9—C52	1.149 (6)	C33—H33A	0.9300
C1—C6	1.362 (8)	C34—C35	1.433 (12)
C1—C2	1.378 (8)	C34—H34A	0.9300
C2—C3	1.412 (9)	C35—C36	1.326 (11)
C2—H2A	0.9300	C35—H35A	0.9300
C3—C4	1.342 (10)	C36—C37	1.412 (9)
C3—H3A	0.9300	C36—H36A	0.9300
C4—C5	1.333 (11)	C37—H37A	0.9300
C4—H4A	0.9300	C38—C39	1.390 (8)
C5—C6	1.401 (9)	C38—C43	1.390 (7)
C5—H5A	0.9300	C39—C40	1.385 (8)
C6—H6A	0.9300	C39—H39A	0.9300
C7—C8	1.349 (8)	C40—C41	1.372 (9)
C7—C12	1.390 (8)	C40—H40A	0.9300
C8—C9	1.393 (10)	C41—C42	1.397 (10)
C8—H8A	0.9300	C41—H41A	0.9300
C9—C10	1.283 (12)	C42—C43	1.356 (9)
C9—H9A	0.9300	C42—H42A	0.9300
C10—C11	1.385 (12)	C43—H43A	0.9300
C10—H10A	0.9300	Cl1A—C53A	1.57 (2)
C11—C12	1.398 (10)	Cl2A—C53A	1.58 (2)
C11—H11A	0.9300	Cl3A—C53A	1.58 (2)
C12—H12A	0.9300	C53A—H53A	0.9800
C13—H13A	0.9700	Cl1B—C53B	1.721 (17)
C13—H13B	0.9700	Cl2B—C53B	1.720 (16)
C14—C15	1.375 (7)	Cl3B—C53B	1.699 (16)
C14—C19	1.376 (7)	C53B—H53B	0.9800
			
C45—Ru1—C44	89.8 (3)	C14—C15—C16	119.4 (6)
C45—Ru1—C46	92.9 (3)	C14—C15—H15A	120.3
C44—Ru1—C46	176.3 (2)	C16—C15—H15A	120.3
C45—Ru1—As1	100.9 (2)	C17—C16—C15	121.0 (6)
C44—Ru1—As1	94.12 (17)	C17—C16—H16A	119.5
C46—Ru1—As1	87.98 (17)	C15—C16—H16A	119.5
C45—Ru1—Ru2	167.2 (2)	C16—C17—C18	120.2 (6)
C44—Ru1—Ru2	93.30 (15)	C16—C17—H17A	119.9
C46—Ru1—Ru2	83.58 (18)	C18—C17—H17A	119.9
As1—Ru1—Ru2	91.263 (18)	C19—C18—C17	118.5 (6)
C45—Ru1—Ru3	109.7 (2)	C19—C18—H18A	120.7
C44—Ru1—Ru3	76.52 (17)	C17—C18—H18A	120.7
C46—Ru1—Ru3	100.10 (17)	C14—C19—C18	120.9 (6)
As1—Ru1—Ru3	147.83 (2)	C14—C19—H19A	119.5
Ru2—Ru1—Ru3	59.268 (13)	C18—C19—H19A	119.5
C48—Ru2—C49	92.4 (3)	C25—C20—C21	119.1 (5)
C48—Ru2—C47	91.4 (3)	C25—C20—As2	123.1 (4)
C49—Ru2—C47	175.0 (2)	C21—C20—As2	117.7 (4)
C48—Ru2—As2	102.07 (17)	C20—C21—C22	119.9 (5)
C49—Ru2—As2	89.41 (16)	C20—C21—H21A	120.0
C47—Ru2—As2	93.03 (16)	C22—C21—H21A	120.0
C48—Ru2—Ru3	100.77 (17)	C23—C22—C21	119.6 (6)
C49—Ru2—Ru3	92.41 (15)	C23—C22—H22A	120.2
C47—Ru2—Ru3	83.63 (15)	C21—C22—H22A	120.2
As2—Ru2—Ru3	156.99 (2)	C24—C23—C22	120.1 (5)
C48—Ru2—Ru1	160.77 (17)	C24—C23—H23A	120.0
C49—Ru2—Ru1	82.88 (15)	C22—C23—H23A	120.0
C47—Ru2—Ru1	92.46 (17)	C23—C24—C25	119.5 (5)
As2—Ru2—Ru1	96.525 (18)	C23—C24—H24A	120.2
Ru3—Ru2—Ru1	61.024 (13)	C25—C24—H24A	120.2
C51—Ru3—C52	101.4 (2)	C20—C25—C24	121.8 (5)
C51—Ru3—C50	92.2 (2)	C20—C25—H25A	119.1
C52—Ru3—C50	166.3 (2)	C24—C25—H25A	119.1
C51—Ru3—P1	94.43 (16)	C27—C26—C31	117.7 (5)
C52—Ru3—P1	89.15 (16)	C27—C26—P1	118.6 (4)
C50—Ru3—P1	91.29 (16)	C31—C26—P1	123.6 (5)
C51—Ru3—Ru2	86.79 (16)	C26—C27—C28	120.3 (6)
C52—Ru3—Ru2	94.01 (15)	C26—C27—H27A	119.8
C50—Ru3—Ru2	85.20 (16)	C28—C27—H27A	119.8
P1—Ru3—Ru2	176.33 (4)	C29—C28—C27	118.9 (7)
C51—Ru3—Ru1	143.46 (16)	C29—C28—H28A	120.5
C52—Ru3—Ru1	69.60 (16)	C27—C28—H28A	120.5
C50—Ru3—Ru1	98.52 (18)	C30—C29—C28	122.1 (6)
P1—Ru3—Ru1	119.95 (4)	C30—C29—Br1	119.3 (6)
Ru2—Ru3—Ru1	59.708 (13)	C28—C29—Br1	118.6 (6)
C7—As1—C1	100.8 (2)	C29—C30—C31	119.1 (7)
C7—As1—C13	104.7 (2)	C29—C30—H30A	120.4
C1—As1—C13	99.0 (2)	C31—C30—H30A	120.4
C7—As1—Ru1	118.55 (17)	C30—C31—C26	121.7 (7)
C1—As1—Ru1	117.51 (16)	C30—C31—H31A	119.1
C13—As1—Ru1	113.54 (15)	C26—C31—H31A	119.1
C20—As2—C14	102.0 (2)	C33—C32—C37	117.9 (6)
C20—As2—C13	104.0 (2)	C33—C32—P1	118.6 (5)
C14—As2—C13	101.7 (2)	C37—C32—P1	123.3 (5)
C20—As2—Ru2	116.18 (14)	C32—C33—C34	121.7 (7)
C14—As2—Ru2	117.40 (15)	C32—C33—H33A	119.2
C13—As2—Ru2	113.62 (14)	C34—C33—H33A	119.2
C38—P1—C32	101.1 (3)	C33—C34—C35	119.4 (7)
C38—P1—C26	101.9 (3)	C33—C34—H34A	120.3
C32—P1—C26	104.9 (2)	C35—C34—H34A	120.3
C38—P1—Ru3	117.08 (17)	C36—C35—C34	119.1 (7)
C32—P1—Ru3	112.92 (17)	C36—C35—H35A	120.4
C26—P1—Ru3	117.05 (18)	C34—C35—H35A	120.4
C6—C1—C2	119.0 (5)	C35—C36—C37	121.2 (8)
C6—C1—As1	120.3 (5)	C35—C36—H36A	119.4
C2—C1—As1	120.7 (4)	C37—C36—H36A	119.4
C1—C2—C3	119.9 (6)	C32—C37—C36	120.7 (7)
C1—C2—H2A	120.0	C32—C37—H37A	119.7
C3—C2—H2A	120.0	C36—C37—H37A	119.7
C4—C3—C2	119.5 (7)	C39—C38—C43	117.3 (5)
C4—C3—H3A	120.2	C39—C38—P1	121.3 (4)
C2—C3—H3A	120.2	C43—C38—P1	121.3 (4)
C5—C4—C3	120.9 (7)	C40—C39—C38	121.4 (5)
C5—C4—H4A	119.6	C40—C39—H39A	119.3
C3—C4—H4A	119.6	C38—C39—H39A	119.3
C4—C5—C6	121.0 (7)	C41—C40—C39	119.4 (6)
C4—C5—H5A	119.5	C41—C40—H40A	120.3
C6—C5—H5A	119.5	C39—C40—H40A	120.3
C1—C6—C5	119.6 (6)	C40—C41—C42	120.1 (6)
C1—C6—H6A	120.2	C40—C41—H41A	119.9
C5—C6—H6A	120.2	C42—C41—H41A	119.9
C8—C7—C12	118.3 (6)	C43—C42—C41	119.3 (6)
C8—C7—As1	123.9 (5)	C43—C42—H42A	120.4
C12—C7—As1	117.7 (5)	C41—C42—H42A	120.4
C7—C8—C9	119.7 (7)	C42—C43—C38	122.3 (6)
C7—C8—H8A	120.1	C42—C43—H43A	118.8
C9—C8—H8A	120.1	C38—C43—H43A	118.8
C10—C9—C8	123.0 (9)	O1—C44—Ru1	171.8 (5)
C10—C9—H9A	118.5	O2—C45—Ru1	178.8 (7)
C8—C9—H9A	118.5	O3—C46—Ru1	173.6 (6)
C9—C10—C11	119.9 (8)	O4—C47—Ru2	174.7 (5)
C9—C10—H10A	120.0	O5—C48—Ru2	177.5 (6)
C11—C10—H10A	120.0	O6—C49—Ru2	174.3 (5)
C10—C11—C12	118.7 (7)	O7—C50—Ru3	175.4 (5)
C10—C11—H11A	120.7	O8—C51—Ru3	176.1 (5)
C12—C11—H11A	120.7	O9—C52—Ru3	169.5 (5)
C7—C12—C11	120.2 (7)	Cl1A—C53A—Cl3A	111 (2)
C7—C12—H12A	119.9	Cl1A—C53A—Cl2A	109 (2)
C11—C12—H12A	119.9	Cl3A—C53A—Cl2A	127 (2)
As1—C13—As2	112.8 (2)	Cl1A—C53A—H53A	102.5
As1—C13—H13A	109.0	Cl3A—C53A—H53A	102.5
As2—C13—H13A	109.0	Cl2A—C53A—H53A	102.5
As1—C13—H13B	109.0	Cl3B—C53B—Cl2B	112.6 (15)
As2—C13—H13B	109.0	Cl3B—C53B—Cl1B	116.8 (16)
H13A—C13—H13B	107.8	Cl2B—C53B—Cl1B	117.9 (14)
C15—C14—C19	119.9 (5)	Cl3B—C53B—H53B	102.0
C15—C14—As2	119.7 (4)	Cl2B—C53B—H53B	102.0
C19—C14—As2	120.4 (4)	Cl1B—C53B—H53B	102.0
			
C45—Ru1—Ru2—C48	−51.6 (12)	C13—As1—C1—C6	95.6 (6)
C44—Ru1—Ru2—C48	52.1 (6)	Ru1—As1—C1—C6	−27.0 (6)
C46—Ru1—Ru2—C48	−125.9 (6)	C7—As1—C1—C2	24.0 (6)
As1—Ru1—Ru2—C48	146.3 (6)	C13—As1—C1—C2	−83.0 (6)
Ru3—Ru1—Ru2—C48	−20.1 (6)	Ru1—As1—C1—C2	154.4 (5)
C45—Ru1—Ru2—C49	−128.2 (10)	C6—C1—C2—C3	−1.2 (11)
C44—Ru1—Ru2—C49	−24.6 (2)	As1—C1—C2—C3	177.4 (6)
C46—Ru1—Ru2—C49	157.5 (2)	C1—C2—C3—C4	0.1 (13)
As1—Ru1—Ru2—C49	69.63 (17)	C2—C3—C4—C5	0.0 (15)
Ru3—Ru1—Ru2—C49	−96.76 (17)	C3—C4—C5—C6	0.8 (16)
C45—Ru1—Ru2—C47	49.9 (10)	C2—C1—C6—C5	2.1 (12)
C44—Ru1—Ru2—C47	153.5 (2)	As1—C1—C6—C5	−176.5 (7)
C46—Ru1—Ru2—C47	−24.5 (2)	C4—C5—C6—C1	−1.9 (15)
As1—Ru1—Ru2—C47	−112.29 (16)	C1—As1—C7—C8	−107.4 (6)
Ru3—Ru1—Ru2—C47	81.33 (16)	C13—As1—C7—C8	−5.0 (6)
C45—Ru1—Ru2—As2	143.2 (10)	Ru1—As1—C7—C8	122.8 (6)
C44—Ru1—Ru2—As2	−113.15 (17)	C1—As1—C7—C12	71.4 (5)
C46—Ru1—Ru2—As2	68.87 (17)	C13—As1—C7—C12	173.8 (5)
As1—Ru1—Ru2—As2	−18.95 (2)	Ru1—As1—C7—C12	−58.3 (5)
Ru3—Ru1—Ru2—As2	174.66 (2)	C12—C7—C8—C9	−0.3 (12)
C45—Ru1—Ru2—Ru3	−31.5 (10)	As1—C7—C8—C9	178.5 (7)
C44—Ru1—Ru2—Ru3	72.19 (17)	C7—C8—C9—C10	−0.5 (16)
C46—Ru1—Ru2—Ru3	−105.79 (17)	C8—C9—C10—C11	0.3 (17)
As1—Ru1—Ru2—Ru3	166.39 (2)	C9—C10—C11—C12	0.8 (15)
C48—Ru2—Ru3—C51	−21.9 (3)	C8—C7—C12—C11	1.4 (10)
C49—Ru2—Ru3—C51	−114.8 (2)	As1—C7—C12—C11	−177.5 (6)
C47—Ru2—Ru3—C51	68.3 (2)	C10—C11—C12—C7	−1.6 (12)
As2—Ru2—Ru3—C51	151.05 (17)	C7—As1—C13—As2	92.9 (3)
Ru1—Ru2—Ru3—C51	164.73 (17)	C1—As1—C13—As2	−163.3 (3)
C48—Ru2—Ru3—C52	−123.1 (3)	Ru1—As1—C13—As2	−37.9 (3)
C49—Ru2—Ru3—C52	144.0 (2)	C20—As2—C13—As1	−107.9 (3)
C47—Ru2—Ru3—C52	−32.8 (2)	C14—As2—C13—As1	146.4 (3)
As2—Ru2—Ru3—C52	49.86 (17)	Ru2—As2—C13—As1	19.3 (3)
Ru1—Ru2—Ru3—C52	63.54 (17)	C20—As2—C14—C15	−164.4 (5)
C48—Ru2—Ru3—C50	70.6 (3)	C13—As2—C14—C15	−57.1 (5)
C49—Ru2—Ru3—C50	−22.3 (2)	Ru2—As2—C14—C15	67.5 (5)
C47—Ru2—Ru3—C50	160.8 (2)	C20—As2—C14—C19	17.8 (5)
As2—Ru2—Ru3—C50	−116.46 (19)	C13—As2—C14—C19	125.1 (5)
Ru1—Ru2—Ru3—C50	−102.79 (18)	Ru2—As2—C14—C19	−110.3 (4)
C48—Ru2—Ru3—Ru1	173.4 (2)	C19—C14—C15—C16	−0.1 (10)
C49—Ru2—Ru3—Ru1	80.49 (16)	As2—C14—C15—C16	−177.9 (5)
C47—Ru2—Ru3—Ru1	−96.39 (17)	C14—C15—C16—C17	−0.3 (11)
As2—Ru2—Ru3—Ru1	−13.68 (5)	C15—C16—C17—C18	0.4 (12)
C45—Ru1—Ru3—C51	146.7 (4)	C16—C17—C18—C19	−0.1 (11)
C44—Ru1—Ru3—C51	−128.4 (3)	C15—C14—C19—C18	0.3 (9)
C46—Ru1—Ru3—C51	50.0 (3)	As2—C14—C19—C18	178.2 (5)
As1—Ru1—Ru3—C51	−52.4 (3)	C17—C18—C19—C14	−0.3 (10)
Ru2—Ru1—Ru3—C51	−26.2 (3)	C14—As2—C20—C25	74.7 (5)
C45—Ru1—Ru3—C52	65.3 (3)	C13—As2—C20—C25	−30.7 (5)
C44—Ru1—Ru3—C52	150.1 (2)	Ru2—As2—C20—C25	−156.4 (4)
C46—Ru1—Ru3—C52	−31.4 (3)	C14—As2—C20—C21	−102.1 (5)
As1—Ru1—Ru3—C52	−133.90 (18)	C13—As2—C20—C21	152.5 (4)
Ru2—Ru1—Ru3—C52	−107.67 (17)	Ru2—As2—C20—C21	26.8 (5)
C45—Ru1—Ru3—C50	−107.7 (3)	C25—C20—C21—C22	1.0 (9)
C44—Ru1—Ru3—C50	−22.9 (2)	As2—C20—C21—C22	177.9 (5)
C46—Ru1—Ru3—C50	155.5 (2)	C20—C21—C22—C23	−0.9 (11)
As1—Ru1—Ru3—C50	53.08 (17)	C21—C22—C23—C24	0.6 (11)
Ru2—Ru1—Ru3—C50	79.31 (16)	C22—C23—C24—C25	−0.3 (10)
C45—Ru1—Ru3—P1	−11.3 (3)	C21—C20—C25—C24	−0.8 (8)
C44—Ru1—Ru3—P1	73.58 (16)	As2—C20—C25—C24	−177.5 (4)
C46—Ru1—Ru3—P1	−107.99 (19)	C23—C24—C25—C20	0.5 (9)
As1—Ru1—Ru3—P1	149.55 (6)	C38—P1—C26—C27	179.3 (5)
Ru2—Ru1—Ru3—P1	175.78 (5)	C32—P1—C26—C27	74.3 (5)
C45—Ru1—Ru3—Ru2	173.0 (3)	Ru3—P1—C26—C27	−51.7 (5)
C44—Ru1—Ru3—Ru2	−102.20 (16)	C38—P1—C26—C31	−1.9 (5)
C46—Ru1—Ru3—Ru2	76.23 (18)	C32—P1—C26—C31	−106.8 (5)
As1—Ru1—Ru3—Ru2	−26.23 (4)	Ru3—P1—C26—C31	127.2 (5)
C45—Ru1—As1—C7	94.2 (3)	C31—C26—C27—C28	3.0 (9)
C44—Ru1—As1—C7	3.7 (2)	P1—C26—C27—C28	−178.1 (5)
C46—Ru1—As1—C7	−173.3 (3)	C26—C27—C28—C29	−2.5 (10)
Ru2—Ru1—As1—C7	−89.72 (18)	C27—C28—C29—C30	−0.6 (11)
Ru3—Ru1—As1—C7	−67.39 (19)	C27—C28—C29—Br1	178.0 (5)
C45—Ru1—As1—C1	−27.4 (3)	C28—C29—C30—C31	3.1 (11)
C44—Ru1—As1—C1	−118.0 (2)	Br1—C29—C30—C31	−175.5 (5)
C46—Ru1—As1—C1	65.1 (3)	C29—C30—C31—C26	−2.5 (11)
Ru2—Ru1—As1—C1	148.64 (19)	C27—C26—C31—C30	−0.5 (9)
Ru3—Ru1—As1—C1	170.97 (19)	P1—C26—C31—C30	−179.3 (5)
C45—Ru1—As1—C13	−142.2 (3)	C38—P1—C32—C33	64.5 (5)
C44—Ru1—As1—C13	127.2 (2)	C26—P1—C32—C33	170.1 (5)
C46—Ru1—As1—C13	−49.7 (2)	Ru3—P1—C32—C33	−61.4 (5)
Ru2—Ru1—As1—C13	33.83 (16)	C38—P1—C32—C37	−119.7 (5)
Ru3—Ru1—As1—C13	56.16 (17)	C26—P1—C32—C37	−14.1 (6)
C48—Ru2—As2—C20	−50.9 (3)	Ru3—P1—C32—C37	114.4 (5)
C49—Ru2—As2—C20	41.4 (2)	C37—C32—C33—C34	0.6 (10)
C47—Ru2—As2—C20	−143.0 (2)	P1—C32—C33—C34	176.6 (5)
Ru3—Ru2—As2—C20	136.14 (17)	C32—C33—C34—C35	−0.3 (11)
Ru1—Ru2—As2—C20	124.12 (16)	C33—C34—C35—C36	−0.1 (12)
C48—Ru2—As2—C14	70.0 (3)	C34—C35—C36—C37	0.3 (13)
C49—Ru2—As2—C14	162.3 (2)	C33—C32—C37—C36	−0.4 (10)
C47—Ru2—As2—C14	−22.1 (2)	P1—C32—C37—C36	−176.2 (5)
Ru3—Ru2—As2—C14	−102.88 (17)	C35—C36—C37—C32	−0.1 (12)
Ru1—Ru2—As2—C14	−114.89 (17)	C32—P1—C38—C39	−149.9 (5)
C48—Ru2—As2—C13	−171.6 (3)	C26—P1—C38—C39	102.2 (5)
C49—Ru2—As2—C13	−79.3 (2)	Ru3—P1—C38—C39	−26.8 (5)
C47—Ru2—As2—C13	96.3 (2)	C32—P1—C38—C43	33.6 (5)
Ru3—Ru2—As2—C13	15.51 (18)	C26—P1—C38—C43	−74.4 (5)
Ru1—Ru2—As2—C13	3.49 (17)	Ru3—P1—C38—C43	156.6 (4)
C51—Ru3—P1—C38	−38.5 (3)	C43—C38—C39—C40	2.2 (9)
C52—Ru3—P1—C38	62.9 (3)	P1—C38—C39—C40	−174.5 (5)
C50—Ru3—P1—C38	−130.8 (3)	C38—C39—C40—C41	0.0 (10)
Ru1—Ru3—P1—C38	128.6 (2)	C39—C40—C41—C42	−3.2 (11)
C51—Ru3—P1—C32	78.3 (3)	C40—C41—C42—C43	4.2 (11)
C52—Ru3—P1—C32	179.6 (3)	C41—C42—C43—C38	−2.0 (11)
C50—Ru3—P1—C32	−14.1 (3)	C39—C38—C43—C42	−1.1 (9)
Ru1—Ru3—P1—C32	−114.7 (2)	P1—C38—C43—C42	175.5 (5)
C51—Ru3—P1—C26	−159.8 (3)	C51—Ru3—C52—O9	60 (3)
C52—Ru3—P1—C26	−58.5 (3)	C50—Ru3—C52—O9	−127 (3)
C50—Ru3—P1—C26	107.8 (3)	P1—Ru3—C52—O9	−35 (3)
Ru1—Ru3—P1—C26	7.2 (2)	Ru2—Ru3—C52—O9	147 (3)
C7—As1—C1—C6	−157.4 (6)	Ru1—Ru3—C52—O9	−157 (3)

### Hydrogen-bond geometry (Å, °)

**Table:**

*D*—H···*A*	*D*—H	H···*A*	*D*···*A*	*D*—H···*A*
C23—H23A···O2^i^	0.93	2.59	3.180 (8)	122

Symmetry codes: (i) *x*−1, *y*, *z*.
